# Delay of cell growth and loss of stemness by inhibition of reverse transcription in human mesenchymal stem cells derived from dental tissue

**DOI:** 10.1080/19768354.2019.1651767

**Published:** 2019-08-13

**Authors:** Won-Cheol Lee, Dae-Young Kim, Mi-Jeong Kim, Hyeon-Jeong Lee, Dinesh Bharti, Sung-Ho Lee, Young-Hoon Kang, Gyu-Jin Rho, Byeong-Gyun Jeon

**Affiliations:** a Department of Biology Education, Gyeongsang National University, Jinju, Republic of Korea; b OBS/Theriogenology and Biotechnology, Gyeongsang National University, Jinju, Republic of Korea; c Division of Life Science, Gyeongsang National University, Jinju, Republic of Korea; d Department of Oral and Maxillofacial Surgery, Changwon Gyeongsang National University Hospital, Changwon, Republic of Korea; e Institute of Education, Gyeongsang National University, Jinju, Republic of Korea

**Keywords:** Human MSCs, reverse transcriptase, nevirapine, cell growth, differentiation

## Abstract

The present study investigated the cellular properties in the dental tissue-derived mesenchymal stem cells (DSCs) exposed to nevirapine (NVP), an inhibitor of reverse transcriptase (RTase). After a prolonged exposure of DSCs for 2 weeks, the population doubling time (PDT) was significantly (*P* < .05) increased by delayed cell growth in the DSCs treated with 250 and 500 μM NVP, compared with untreated DSCs. Furthermore, the G1 phase of cell cycle with high activity of senescence-associated β-galactosidase was also significantly (*P* < .05) increased in the 250 μM NVP-treated DSCs, compared with untreated DSCs. The level of telomerase activity was unchanged between control and treatment. However, following the treatment of NVP, negative surface markers for mesenchymal stem cells (MSCs), such as CD34 and CD45, were significantly (*P* < .05) increased, while positive surface markers for MSCs, such as CD90 and CD105, were significantly (*P* < .05) decreased in the NVP-treated DSCs than those of untreated DSCs. Furthermore, the differentiation capacity into mesodermal lineage was gradually decreased, and a significant (*P* < .05) decrease of expression level of NANOG, OCT-4 and SOX-2 transcripts was observed in the DSCs treated with NVP, compared with untreated control DSCs. Taken together, the present results have revealed that inhibition of RTase by NVP induces delayed cell growth and loss of stemness.

## Introduction

Approximately 1.5% of human genome with 23 chromosome pairs is only covered with coding sequences that can be translated to proteins. But, over 98% of the genome consisted of non-coding sequences, including introns, sequences for non-coding RNAs (including rRNA and tRNA), regulatory regions (promoters, enhancers and so on), and tandem and interspersed repeats. Furthermore, the largest component (∼45%) in the non-coding sequences of the genome is transposable elements (TEs), including Class I (retrotransposons) and Class II (DNA transposons), and most of the TEs are Class I retrotransposons (Mills et al. 2007; Lander 2011). A part of the Class II DNA transposons is severed and transposed at new position of the genome, as ‘cut and paste’ mechanism, while a part of Class I retrotransposons is copied to RNA intermediate, converted into DNA by the activity of a reverse transcriptase (RTase) and transposed, as ‘copy and paste’ mechanism (Magiorkinis et al. 2017). Therefore, the activity of RTase is definitely needed for the incorporation and transposition of retrotransposons into the genome. Since the TEs are capable of jumping and replicating themselves to new locations or regulatory regions in the genome, the sequence and size of the genome can subsequently be altered, rearranged or increased. It has been thus suggested that the TEs may lead to dramatic effects on gene expression, rather than useless junk DNA. Furthermore, many recent studies have increasingly demonstrated that TEs are tightly related with several types of diseases in human beings and contributed to the evolution of gene family as well as gene expression by DNA editing is presently under debate (Kazazian et al. 1988; Pantzartzi et al. 2018). Retroviruses are mostly RNA viruses that convert their RNA genome into the DNA intermediate using their RTase activity (Preston et al. 1988). The human immunodeficiency virus (HIV) is also the retrovirus that causes HIV, and there are several types of RTase inhibitor for the treatment of acquired immune deficiency syndrome (AIDS) and HIV infection. The nucleoside or nucleotide analog, such as zidovudine and didanosine, is used for the inhibition of RTase. Furthermore, nevirapine (NVP) and efavirenz are also a kind of non-nucleoside RTase inhibitors that noncompetitively bind at an active site of the RTase enzyme, and are commonly used as a medication for the treatment of AIDS and HIV infection (Flexner 2018; Gu et al. 2018). Moreover, even though the basic biological functions of RTase activity in the cancer cell lines are still the question, several cancer cells possess the high level of RTase activity and NVP treatment has been induced to the delayed cell growth and cellular differentiation, probably malfunction of retrotransposons by inhibition of RTase activity, and subsequently alteration of epigenetic modification in the cancer cells (Sciamanna et al. 2005; Jeon et al. 2011a).

The adult mesenchymal stem cells (MSCs) derived from bone marrow have been the ideally primary source in regenerative medicine. However, it involves painful isolation procedures and moreover the extracted cell number yield is also very low. On the other hand, MSCs derived from dental tissue of extracted third molar (wisdom tooth) have gained much importance due to their outstanding stemness properties, high cell yield and multidifferentiation potential when compared to other tissues-derived MSCs (Jeon et al. 2011a, 2011b, 2015). In our previous studies, it has also been demonstrated that DSCs, as transformed cancer cell lines, also possess the high activity of non-telomeric RTase that related with transposition of Class II retrotransposons (Jeon et al. 2011a). However, the functions and roles of TEs and RTase activity in the MSCs are still not fully known. The cellular properties, such as the population doubling time (PDT) based on cell growth rate and cell cycle status, have been used as the fundamental markers for cellular characteristics of the isolated MSCs (Jeon et al. 2011b, 2015). And, the telomerase activity related with shortening of the telomeric repeats has shown to be tightly related to the MSCs lifespan and proliferation capacity (Jeon et al. 2011a, 2011c). It has been further suggested that most of the MSCs isolated from various tissues are primarily identified by several defining characteristics. MSCs must exhibit plastic-adherent property on cell culture dish, and highly express cluster of differentiation (CD) markers on the their surface, such as CD105, CD73 and CD90 markers, but lack expression of CD14, CD31, CD34 and CD45 markers. Most importantly, MSCs must display differentiation capacity into mesodermal lineage, such as osteocytes, adipocytes and chondrocytes, and possess the expression of stem cell-specific genes such as *NANOG, OCT-4 and SOX-2* which are essential for their stemness maintenance (Dominici et al. 2006; Calloni et al. 2013; Huang et al. 2015).

To further evaluate the functions of RTase in the MSCs, the isolated dental tissue-derived MSCs (DSCs) were firstly exposed with nevirapine (NVP), a non-nucleoside RTase inhibitor, and we further investigated the effect of NVP on the fundamental cell properties such as PDT with cell cycle status and senescence-associated-ß-galactosidase (SA-ß-gal) activity, and relative telomerase activity in the NVP-treated DSCs. Similarly, NVP-treated DSCs were further assessed for their stemness characterizations, such as CD cell surface markers, differentiation capacity into mesodermal lineage and expression pattern of stem cell-specific genes. Thus, we have tried to investigate the basic functions and roles of RTase and Class II retrotransposons via the comparison of fundamental cellular properties and stemness characterizations after inhibition of RTase with NVP in the MSCs.

## Materials and methods

### Culture and treatment of DSCs

The basic cell culture medium for DSCs was advanced-Dulbecco’s modified eagle medium added with 10% fetal bovine serum (FBS) and 1.0% penicillin–streptomycin (10,000 IU and 10,000 μg/ml, respectively) and was purchased from Gibco (USA). The nevirapine (NVP) was purchased from Tokyo Chemical Industry (Japan). The other chemicals were purchased from Sigma (USA), unless otherwise specified. The dental tissues were collected from papilla and pulp parts of the third molar tooth of three healthy donors, and dental cells were subsequently harvested by Ficoll gradient, as previously described (Jeon et al. 2011b, 2015). The cells were further cultured in a humidified atmosphere of 5% CO_2_, incubator at 37.5°C, and passaged at 80∼90% confluent status by changing the media twice a week. At passage 3, the stem cell-specific characterizations of the isolated cells were also demonstrated as per our previous study and isolated cells were cryopreserved further (Jeon et al. 2011b, 2015). DSCs at passage 5 were used in this study. The NVP was dissolved at 250 mM in dimethyl sulfoxide (DMSO) and freshly prepared basic culture medium was used for NVP treatment.

### Analysis of population doubling time

The effect of growth inhibition was analyzed by the evaluation of PDT assay in the NVP-treated DSCs. Briefly, DSCs at the cell density of 1 × 10^3^ cells/well into a 6-well plate were seeded and cultured in A-DMEM media containing with 0 μg/ml (untreated control), 0.5% DMSO, 100 μM NVP, 250 μM NVP and 500 μM NVP at 37.5°C in a 5% CO_2_ incubator for 2 weeks, respectively, and each culture media were regularly changed every three days. After 2 weeks, the cells were collected by trypsination and the cell number was counted with a hemocytometer. The PDT was calculated by the following formula: PDT = ln (log N_t_/N_o_)/t, where t the is cell culture time between N_o_ and Nt, N_o_ and N_t_ are the initial and final cell numbers, respectively.

### Analysis of cell cycle and CD markers by flow cytometry

With the help of flow cytometry (BD FACSCalibur, Becton Dickinson, USA), cell cycle and CD marker expression were evaluated in 250 μM NVP-treated DSCs for 2 weeks, as previously described (Jeon et al. 2011b, 2015). For the analysis of cell cycle, the untreated control and NVP-treated DSCs at ∼70% confluence were collected by 0.25% trypsin treatment. After being washed with D-PBS, the DSCs were fixed with 70% ethanol, and again washed with D-PBS. The DSCs were stained with 10 mg/ml propidium iodide (PI) solution for 30 min at room temperature. For the analysis of CD markers, the CD 34 and CD45 were used as negative MSC cell surface markers and CD 90 and CD105 as positive MSC markers, respectively. The collected DSCs were also washed with D-PBS and subsequently were fixed with 3.7% formaldehyde solution in D-PBS at 4°C. The labelling of DSCs was performed with a fluorescein isothiocyanate (FITC)-conjugated mouse anti-CD34, CD45 and CD90 (BD, USA, 1:100) for 1 h on ice. While after being incubated with FITC-unconjugated mouse anti-CD105 (Santa Cruz, USA, 1:100) for 45 min at 37°C, the DSCs were subsequently labelled with FITC-conjugated goat anti-mouse IgG (BD, USA) secondary antibody at 4°C for 1 h. The PI and FITC-labelled 1 × 10^5^ DSCs were used for assay using a flow cytometer, and the data were analyzed with CellQuest software in triplicate (Becton Dickinson, USA).

### Analysis of senescence-associated β-galactosidase (SA β-gal) activity

The cellular senescence by NVP treatment was evaluated with the analysis of senescence-associated β-galactosidase (SA β-gal) activity. Cells were seeded at the density of 1 × 10^3^ cells/well into a 6-well plate and cultured in A-DMEM media containing 0 μg/ml (untreated control) and 250 μM NVP, respectively. After 2 weeks, the SA β-gal activity was evaluated with SA β-gal staining kit, as per the manufacturer’s protocol (Cell Signaling Technology, USA). Briefly, each 6-well plate was rinsed with D-PBS, and added with a fixative solution for 10–15 min at room temperature. Each 6-well plate was subsequently added with β-galactosidase staining solution, and then each 6-well sealed with parafilm was incubated at 37°C for overnight. After being washed with D-PBS, the stained cells were evaluated under an inverted microscope (Nikon, Japan) equipped with a CCD camera and image program. Cells that were shown to have blue color exhibited high SA β-gal activity.

### Analysis of relative telomerase activity

The relative telomerase activity was quantified by relative-quantitative telomerase repeat amplification protocol (RQ-TRAP) assay in both the NVP-treated and treated DSCs. The RQ-TRAP assay using a real-time PCR machine (Rotor-Gene Q, Qiagen, USA) was slightly modified from the traditional gel-based original TRAP assay protocol, as previously described by Jeon et al. (2011b). Briefly, after NVP treatment, each sample was harvested at approximately 1 × 10^5^ cells/sample, and added with 400 µl of TRAPeze® 1X CHAPS cell lysis buffer (Millipore, USA) for 30 min on ice. Then lysed samples were centrifuged for 20 min at 12,000 × g at 4°C, and approximately 60–70% (by volume) of the supernatant was selected and transferred to a fresh sample tube. The protein concentration in each sample was subsequently measured with a spectrophotometer (Mecasys, Korea). The each reaction in the 20 μl of final volume consisted of Rotor-Gene^TM^ 2× SYBR green kit (Qiagen, USA), 0.02 µg of telomerase TS primer, 0.04 µg of anchored return ACX primer, and 1 μg of total protein of each sample. The sequences of TS and ACX primer are shown in Table 1. Each reaction was firstly incubated at 30°C for 30 min and then denatured at 94°C for 10 min. The real-time amplification protocol consisted of 94°C for 30 sec, 60°C for 90 sec and 72°C for 0 sec for 40 cycles. After amplification, the difference of crossing point (Cp) was analyzed with Rotor-Gene Q Series Software (Qiagen, USA). The level of relative telomerase activity by NVP treatment was compared with those of untreated human fetal MRC-5 fibroblasts and human A-549 lung adenocarcinoma cells were used as the internal control. Each sample was performed in triplicates.

**Table 1.** Primer sequences and amplification size used for RT-PCR.

Gene	Primer sequences (5′–3′)	Amplification size (bp)
RQ-TRAP TS	AATCCGTCGGAGCAGAGTT	
RQ-TRAP ACX	GCGCGGCTTACCCTTACCCTTACCCTAACC	
NANOG	AGAAGGCCTCAGCACCTAC GGCCTGATTGTTCCAGGATT	205
OCT4	CGACCATCTGCCGCTTTGAG CCCCCTGTCCCCCATTCCTA	577
SOX-2	CCCCCGGCGGCAATAGCA TCGGCGCCGGGGAGATACAT	448
GAPDH	GAAGGTGAAGGTCGGAGTC GAAGATGGTGATGGGATTTC	228

### Analysis of differentiation capacity into mesodermal lineage

The differentiation capacity into mesodermal osteocytes, chondrocytes and adipocytes was analyzed in the both the NVP-treated and untreated DSCs for 4 weeks, as previously described (Jeon et al. 2015). Briefly, the differentiation medium into osteocytes consisted of DMEM supplemented with 1 μM dexamethasone, 10 mM sodium β-glycerophosphate and 0.05 mM ascorbic acid. The accumulation of calcium in the osteocytes was confirmed by Alizarin red staining. The differentiation medium into chondrocytes was 5 ng/ml transforming growth factor-β1, 0.1 μM dexamethasone, 50 mg/ml ascorbic acid, 100 mg/ml sodium pyruvate, 40 mg/ml L-proline and 50 mg/ml ITS + premix (6.25 mg/ml insulin, 6.25 mg/ml transferrin, 6.25 ng/ml selenious acid, 1.25 mg/ml BSA and 5.35 mg/ml linoleic acid). The accumulation of proteoglycan in the chondrocytes was assessed by Alcian blue staining. But, for adipogenic differentiation DMEM was supplemented with 10 μM insulin, 100 μM indomethacin, 500 μM isobutyl methylxanthine and 1 μM dexamethasone. The neutral triglycerides and lipids in the oil droplets of adipocytes were confirmed by Oil red O staining. The degree of cellular differentiation was visually compared by evaluating the intensity of each staining solution under an inverted microscope. Images from all of the stained cells were acquired.

### Analysis of stem cell-specific transcripts

The expression level of stem cell-specific transcripts, such as SOX-2, OCT-4 and NONOG, was analyzed by reverse transcription polymerase chain reaction (RT–PCR) assay in both the NVP-treated and untreated DSCs. Firstly, the total RNA from each sample was extracted with Ribospin extraction kit, as per the manufacturer’s instructions (GeneAll, Korea). After quantifying total RNA with the help of spectrophotometer, 1 μg of total RNA was converted to cDNA with 2 μl of 10 μM random hexamer, 1 μl of 10 U/μl RNase inhibitor, 2 μl of dNTP, 4 U RTase (Qiagen, USA) in a 20 μl each reaction at 42°C for 1 h. Furthermore, the cDNA was amplified by PCR protocol using Maxime-PCR PreMix Kit (iNtRON Biotechnology, Korea). The each amplification reaction in the 20 μl of final volume consisted of 2 μl of cDNA sample, and 1 μl each of the 10 μM forward and reverse primer. The primer sequence for each transcripts is shown in Table 1. The amplification protocol was 30 cycles of denaturation step at 94°C for 1 min, annealing step at 55°C for 30 sec and elongation step at 72°C for 1 min. The PCR products were confirmed on 1% agarose gel, and the images were acquired with E-graph software (ATTO, Japan). The expression level of SOX-2, OCT-4 and NONOG transcript was compared with that of gene glyceraldehyde 3-phosphate dehydrogenase (GAPDH), as internal standard.

### Statistical analysis

All data were represented as mean ± standard error of the mean (mean ± SEM). The significance of the differences in the acquired data over at least three replicates was analyzed by using one-way analysis of variance (ANOVA, SPSS 15.0 version, USA). The level of statistical significance was checked at *P* < .05.

## Results

### Analysis of PDT

**Figure 1.**
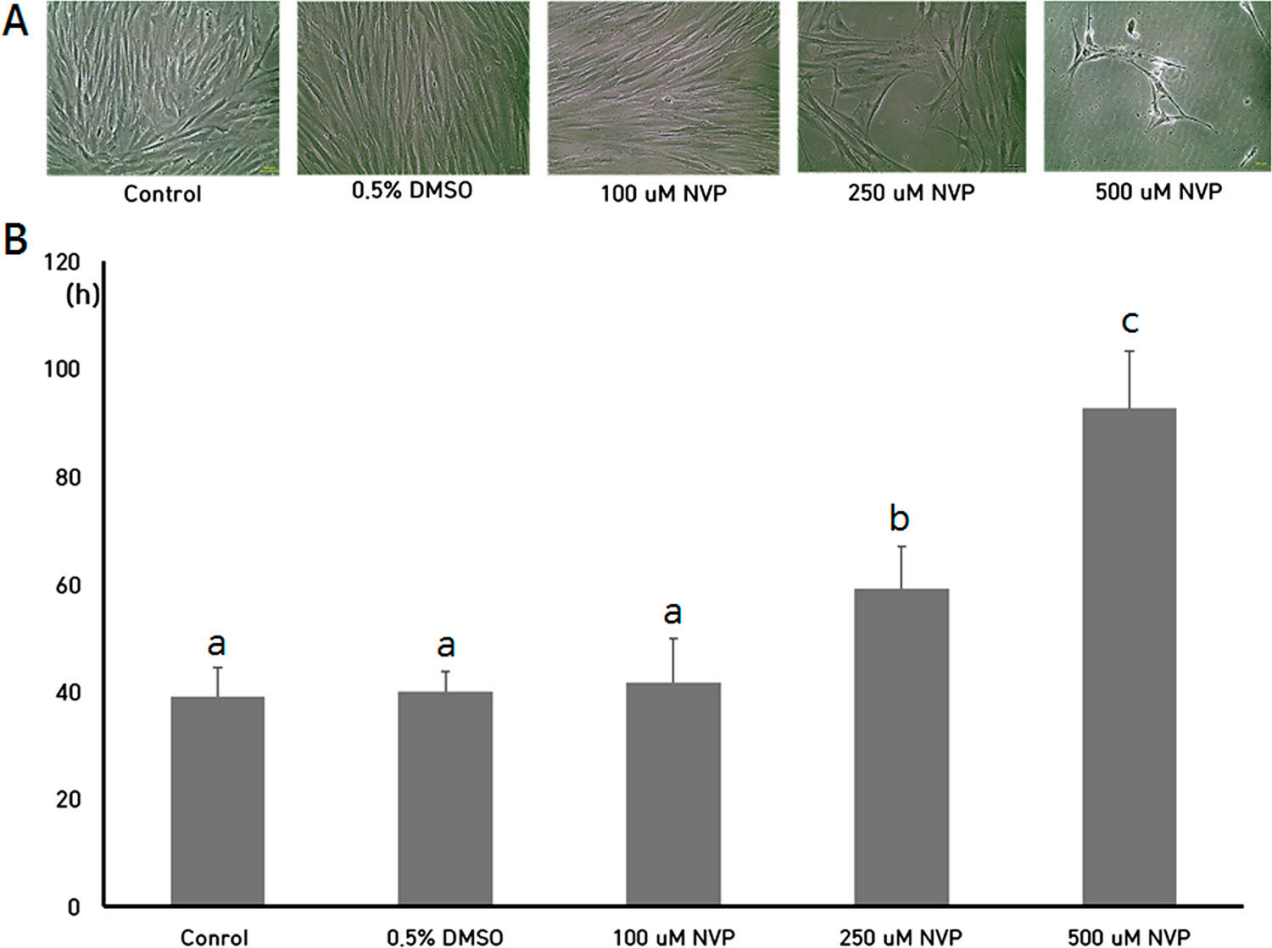
Cell growth (A) and analysis of PDT (B) in DSCs treated with 100, 250 and 500 uM up to 2 weeks. a, b and c indicate significant (*P* < .05) difference among treatments, respectively. Star-like shapes were observed in the DSCs treated with 500 uM NVP. The values in Figure 1(B) indicated mean ± SEM of three replicates in each of three DSCs. Scale: 50 μm. A representative example of three DSCs is shown in Figure 1(A).

After the NVP treatment for 2 weeks, the cell number was counted and PDT of each DSCs was investigated, as shown in Figure 1. The PDT (mean ± SEM) in untreated control DSCs, DSCs supplemented with 0.5% DMSO, DSCs treated with 100 μM NVP, DSCs treated with 250 μM NVP and DSCs treated with 500 μM NVP were 39.2 ± 5.34, 40.1 ± 3.68, 41.7.0 ± 8.23, 59.3 ± 7.79 and 92.7 ± 10.51, respectively. No significant differences were observed in the PDT of untreated control and 0.5% DMSO groups. After the NVP treatment, the PDT was significantly (*P* < .05) increased by the decreased cell proliferation rate in the DSCs treated with 250 and 500 μM NVP, but PDT by treatment of 100 μM NVP was similar to that of the untreated controls.

### Analysis of cell cycle and SA β-gal activity

**Figure 2.**
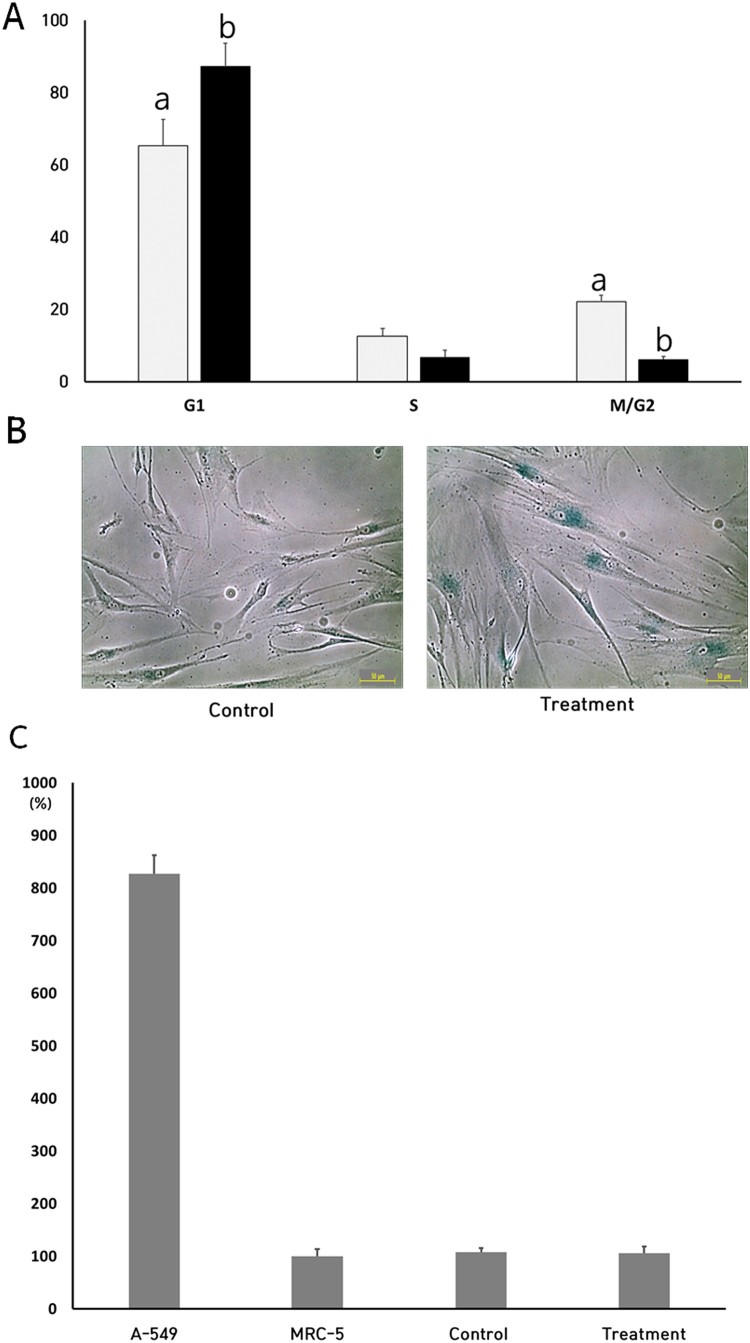
Analysis of cell cycle (A), activity of senescence-associated-β-galactosidase (B) and activity of telomerase in untreated control (▪) and 250 uM NVP treatment (▪) for 2 weeks. a and b indicate significant (*P* < .05) difference between untreated control and NVP treatment, respectively. The values in Figure 2(A,C) indicated mean ± SEM of three replicates in each of three DSCs. Scale: 50 μm. A representative example of three DSCs is shown in Figure 2(B).

After the treatment of 250 μM NVP for up to 2 weeks, the cell cycle and SA β-gal activity were measured, as shown in Figure 2(A,B). The proportions of cells in G1, S and G2/M phase were 65.3 ± 7.34, 12.6 ± 2.13 and 22.1 ± 1.83% in the untreated control DSCs, respectively. But, the proportion of cells in the G1, S and G2/M phase was 87.2 ± 6.46, 6.7 ± 1.95 and 6.1 ± 0.87% in the NVP-treated DSCs, respectively. Especially, the G1 phase of cell cycle was significantly (*P* < .05) increased by the NVP treatment. Furthermore, the cell morphology was gradually enlarged, and the frequency of cells with high activity of SA β-gal was also increased in the NVP-treated DSCs.

### Analysis of telomerase activity

After the treatment of 250 μM NVP for up to 2 weeks, the level of telomerase activity, a kind of RTase, was analyzed by RQ-TRAP assay, as shown in Figure 2C. A-549 cells, which have high replicative ability showed a high level of telomerase activity, were used as an internal control. These cells were expressed at ∼ 8-fold in comparison to untreated normal MRC-5 fibroblasts, and the level of telomerase activity in the untreated DSCs was 108 ± 8.2%, when compared with normal MRC-5 fibroblasts. Furthermore, the level of telomerase activity was 106 ± 12.4 in the NVP-treated DSCs. The level of telomerase activity tended to be slightly increased between the untreated and NVP-treated DSCs, but was not significantly (*P* < .05) different.

### Analysis of cell surface markers

**Figure 3.**
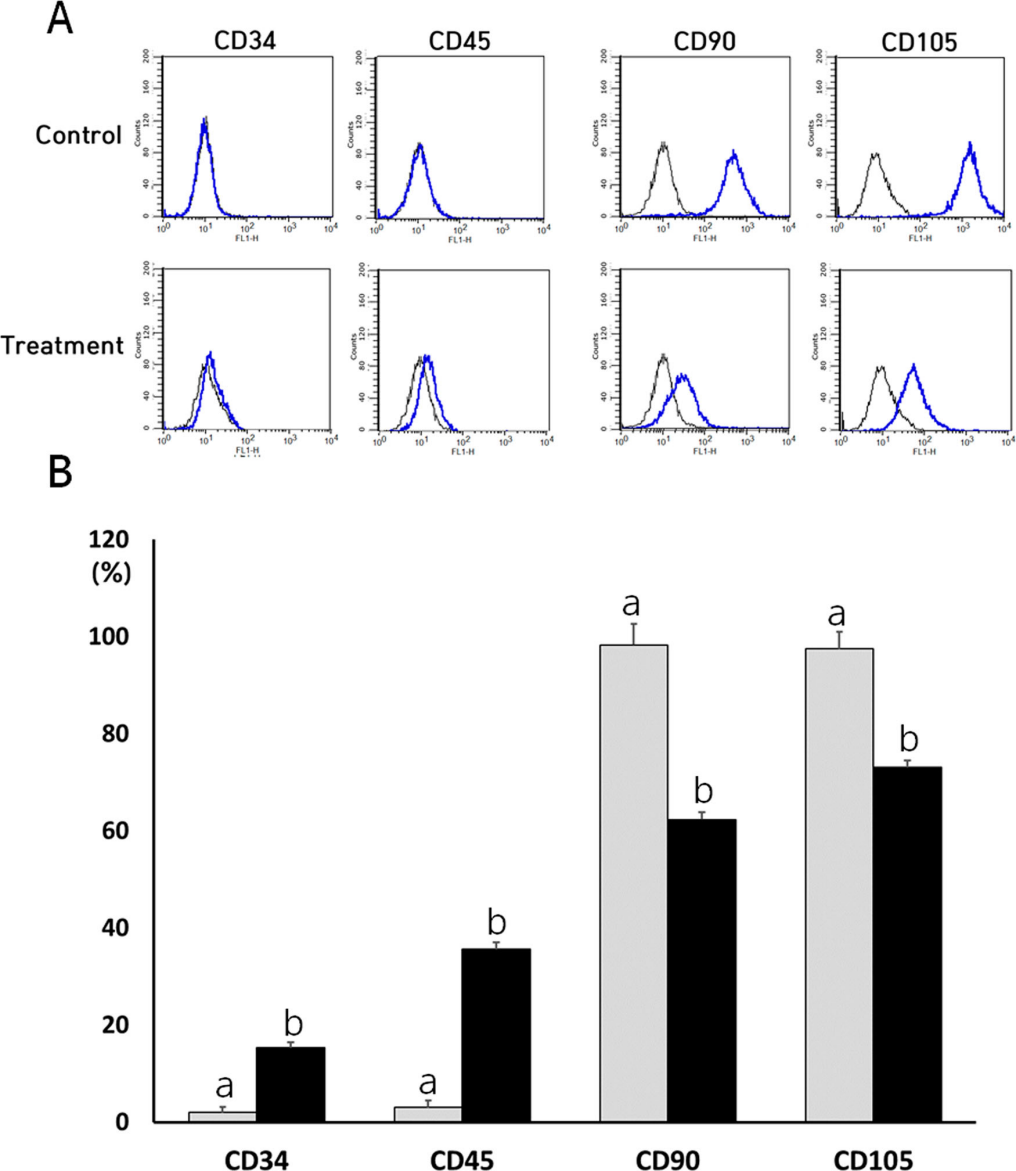
Representative images by cytometric analysis of cell surface markers (A) and expression analysis of cell surface markers in untreated control (▪) and 250 uM NVP treatment (▪) for 2 weeks. CD34 and CD45 were used as negative marker for MSCs, and whereas CD90 and CD105 were used as positive marker for MSCs, respectively. a and b indicate significant (*P* < .05) difference between untreated control and NVP treatment, respectively. A representative example of three DSCs is shown in Figure 3(A). The values in Figure 3(B) indicated mean ± SEM of three replicates in each of three DSCs.

Both the NVP-treated and untreated control DSCs were evaluated for the expression of cell surface markers with the help of flow cytometry (Figure 3). Untreated control DSCs were shown to have low expression of CD34 and CD45 markers with the expression values of 2 ± 1.0 and 3 ± 1.4%, respectively. But, the expression level of CD34 and CD45 was 15 ± 1.2 and 35.6 ± 1.5% in the NVP-treated DSCs, respectively. The expression of CD34 and CD45 MSCs-negative surface markers was significantly (*P* < .05) increased after the NVP treatment. In addition, untreated control DSCs showed high expression levels for CD90 (98 ± 1.5%) and CD105 (97 ± 1.5%) markers. However, the expression level of CD90 and CD105 was 62 ± 4.4 and 73 ± 3.5% in the NVP-treated DSCs, respectively. The NVP treatment resulted in significantly decreased (*P* < .05) expression for CD90 and CD105.

### Analysis of differentiation capacity into mesodermal lineage

**Figure 4.**
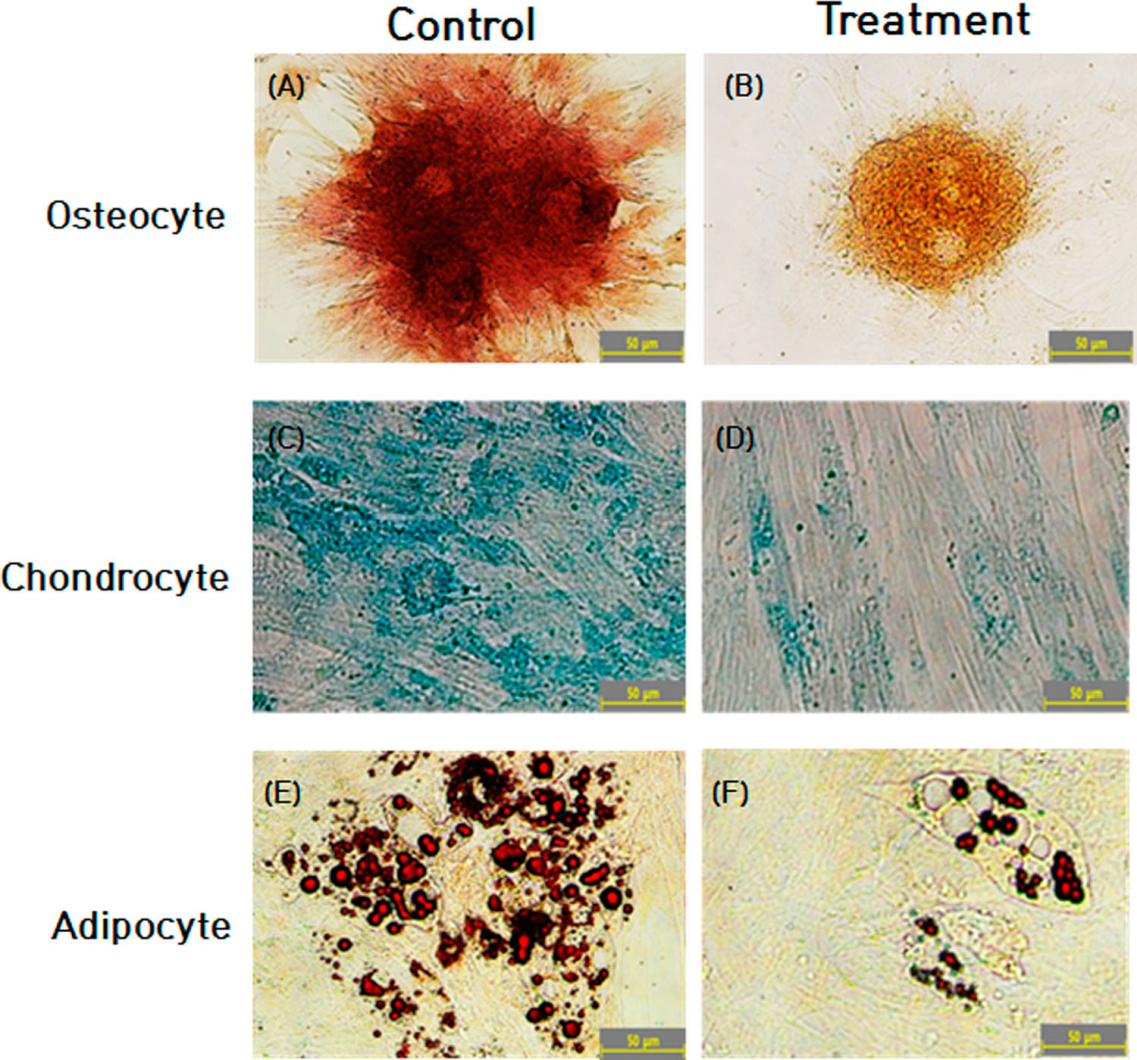
*In vitro* differentiation capacity into osteocyte (A and B), chondrocyte (C and D) and adipocyte (E and F) in the untreated control (A, C and E) and 250 uM NVP treatment (B, D and F). Intracellular accumulation of calcium was observed by Alizarin red staining (B) in osteocyte (A and B). Synthesis of glycosaminoglycans was observed by Alcian blue staining in chondrocyte (C and D). Intracellular accumulation of lipids was revealed by Oil red O staining in adipocyte (E and F). A representative example of three DSCs is shown in Figure 4.

Following the NVP treatment for up to 2 weeks, the differentiation capacity into mesodermal lineage, such as osteocytes, chondrocytes and adipocytes was evaluated by inducing the cells under lineage-specific differentiation conditions. The intensity of Alizarin red staining for the osteocyte and Alcian blue staining for the chondrocytes was strongly expressed, and oil pellets with deeper red color by Oil red O staining in the adipocytes were highly observed in the untreated control DSCs. Thus, the untreated control DSCs were observed to be readily differentiated into mesodermal lineage, as shown in Figure 4. But, NVP-treated DSCs showed decreased differentiation ability into adipocyte, osteocyte and chondrocyte lineages, as evaluated by the reduced staining intensity for Oil red O, alizarin red and alcian blue stains. Collectively, differentiation capacity into osteocytes, chondrocytes and adipocytes was found to be gradually decreased after the NVP treatment.

### Expression level of stem cell-specific transcripts

**Figure 5.**
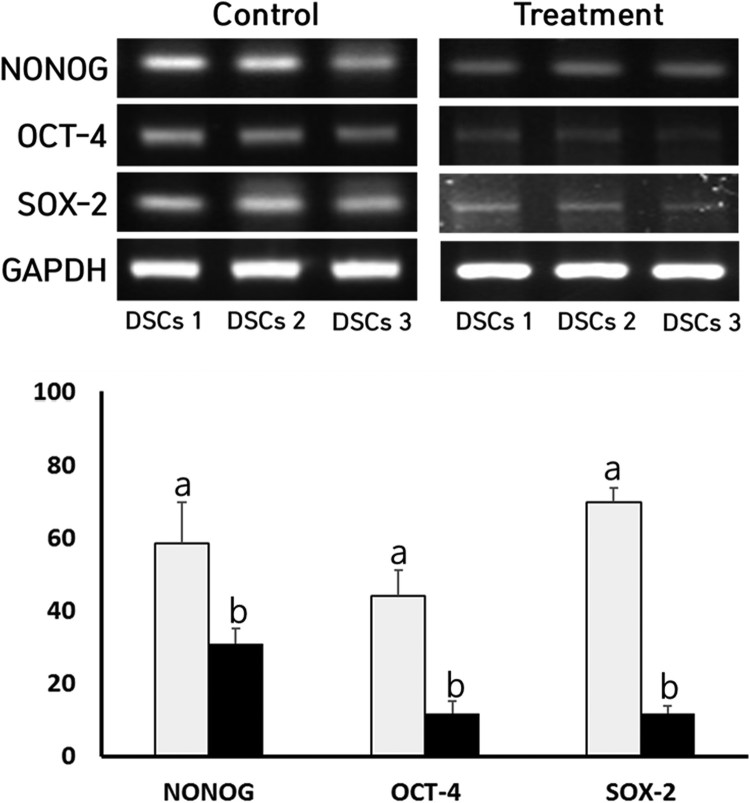
A, Expression level of NANOG, OCT-4 and SOX-2 transcription factors by RT-PCR in untreated control (▪) and 250 uM NVP-treated (▪) DSCs. a and b indicate significant (*P* < .05) difference between untreated control and NVP treatment, respectively.

The relative expression level of stem cell-specific transcripts, such as NONOG, OCT-4 and SOX-2, was investigated by RT–PCR assay in the NVP-treated DSCs and the result is shown in Figure 5. The mean expression level of NONOG, OCT-4 and SOX-2 transcripts was, respectively, 58 ± 11.3, 44 ± 6.9 and 96 ± 4.0% in the untreated control DSCs, as compared with level of GAPDH, an internal control gene. But, the mean expression level of NONOG, OCT-4 and SOX-2 transcripts was 31 ± 4.2, 11.8 ± 3.3 and 11.8 ± 2.1% in the NVP-treated DSCs, respectively. The expression of NONOG, OCT-4 and SOX-2 stem cell-specific transcripts was significantly (*P* < .05) decreased after the NVP treatment.

## Discussion

In the present study, the MSCs derived from dental tissues of wisdom teeth (DSCs) were exposed to nevirapine (NVP) which is commonly used as an antiretroviral drug via inhibition of RTase, for up to 2 weeks. NVP-untreated DSCs were taken as control cells. Then both the NVP-treated and untreated DSCs were investigated for the alteration of their fundamental cellular properties, such as cell proliferation, phase of cell cycle with senescence-associated β-galactosidase (SA β-gal) activity and telomerase activity. All experimental groups were further investigated for the expression of their stemness characterizations, such as CD34, CD45, CD90 and CD105 surface markers for MSCs, cellular differentiation capacity into mesodermal osteocytes, chondrocytes and adipocytes, and stem cell-specific NONOG, OCT-4 and SOX-2 transcription factors. Our results showed that the inhibition of RTase using NVP resulted in the reduction of cell growth with cellular senescence, and further decreased stemness characterizations in the DSCs.

NVP selectively inhibits the cellular activity by distorting the dNTP-binding pocket at the active site of the RTase. Thus, NVP is a kind of non-nucleoside RTase inhibitor for the treatment of patients with acquired immunodeficiency syndrome (AIDS) by the inhibition of endogenous RTase which usually contained in the retrovirus, such as human immunodeficiency virus type 1 (HIV-1) (Flexner 2018). However, the several side effects, such as rash, nausea, fatigue, headache, vomiting, diarrhoea, and pain, have been reported after oral intake of NVP (Chowta et al. 2018). It has been further reported that *in vitro* direct treatment of NVP is induced to the induction of hepatotoxicity by mitochondrial dysfunction and apoptosis in HepG2 cells (Wongtrakul J, et al. 2016; Paemanee et al. 2017). The treatment of NVP has also been demonstrated to delay cell growth in the various types of cells, especially cancer cells. Thus, several types of chemicals with the ability to inhibit RTase have been considered as anti-tumor drugs in the chemotherapeutic treatment of cancer (Mangiacasale et al. 2003; Sinibaldi-Vallebona et al. 2011; Hecht et al. 2015). Previously, one more study has also demonstrated that the NVP treatment could result in the arrest of cell growth and cellular senescence in human cervical carcinoma cells (Stefanidis et al. 2008). Furthermore, the inhibition of RTase by efavirenz, another kind of non-nucleoside RTase inhibitor and RNA interference, has also shown to be selectively down-regulated the proliferation in the transformed cancer cell lines (Sciamanna et al. 2013). Our previous study has also shown that the RT activity is highly expressed in the MSCs as well as in the cancer cells (Jeon et al. 2011b). And our present study utilizing MSCs has shown similar results in accordance with the earlier studies using cancer cells, demonstrating that the NVP treatment is induced to arrest of cell growth in the cancer cells.

Our results have also shown that the NVP treatment is displayed to the increased G1 phase of cell cycle and high level of SA β-gal activity. In accordance with the previous reports, our study has also demonstrated an increase in the G1 phase of cell cycle under the influence of the NVP treatment. The SA β-gal activity is highly expressed in the cells reached at cellular senescent status, and is widely used for the investigation of cellular senescence and damage (Moon et al. 2015; Kim et al. 2017). Previous studies have reported that the cells reached at cellular senescence or delayed cell growth were generally arrested at the G0/G1 phase of cell the cycle (Funayama and Ishikawa 2007; Mao et al. 2012). Therefore, we assume that NVP treatment seems to be induced to cellular senescence by arrest of cell cycle by cell damage and growth inhibition. However, in the present study, the functions and roles of RTase on the cell growth and cellular senescence have not fully demonstrated and still remain uncertain in the MSCs. And further fundamental studies are warranted to investigate the effects in the DSCs after the NVP treatment.

In all eukaryotes, the RNA intermediates that are transcribed from some DNA sequences, referred to as retrotransposons, are converted to complementary DNA (cDNA), responding to the RNA by the activity of reverse transcription. The cDNA can be transposed into a new position of their or host genome (Mills et al. 2007; Lander 2011). The generalized functions and roles of RTase within the cells are mainly responsible for the production of double-stranded complementary DNA (cDNA) from single-stranded RNAs, and incorporation of the cDNA into other DNA site. Thus, it has been increasingly suggested that roles of retrotransposons produced by the activity of RTase are dynamically associated with the modification of gene expression via epigenetics modification, such as altering promoters, regulatory modules and chromatin structure (Cordaux and Batzer 2009; Yin et al. 2018). The cell growth and division are generally controlled by a great diversity of genes which needs to be timely transcribed as per the events of cell cycle. And inhibition of the gene expression related with cell growth is commonly induced to the arrest of cell cycle and growth of the cells (Simmons Kovacs et al. 2008; Müller and Engeland 2010). As shown in the present results, the arrest of cell growth and induction of senescence is also thought to be a consequence of decreased or changed gene expression by epigenetic modification of the cells exposed to NVP. It has been widely demonstrated that telomerase activity is related to cell growth and lifespan in cancer and normal cells (Jeon et al. 2011c; Kim et al. 2017). However, even though cell growth was retarded in the DSCs treated with NVP, present results have shown that the telomerase activity was not changed by the NVP treatment. The telomerase activity is also a kind of RTase responsible for the elongation or maintenance of telomere sequence repeat to the 3′ end of telomeres using telomerase RNA component (TERC) and the high level of telomerase activity was expressed in the immortal cell lines, such as cancers and embryonic stem cells (Weinrich et al. 1997; Artandi and DePinho 2010; Kim et al. 2017). Moreover, the cancer cells under *in vitro* cancer treatment displayed decrease in cell growth and down-regulated telomerase activity (Kim et al. 2017). In most of the MSCs, including DSCs, the previous reports have continually demonstrated that telomerase activity is detected at a basal level; therefore, other mechanisms for maintenance of telomeric repeats in human MSCs (Hiyama and Hiyama 2007; Jeon et al. 2011a, 2011b; Kim et al. 2017, 2018). Since telomerase activity is originally at a basal level in the normal DSCs, we thus assume that arrest of cell growth and cellular senescence is induced by the NVP treatment ; however, telomerase activity might not be down-regulated in the DSCs treated with NVP. Furthermore, even though telomerase activity is also a kind of RTase, it has been suggested that RTase activity is different from telomerase activity (Jeon et al. 2011a, 2011b). In the present study, RTase activity is inhibited by the NVP treatment; however, telomerase activity was not altered, and our result on the telomerase activity might be partially supported that the RTase for transposition of the retrotransposons is endogenous non-telomeric RTase, districting from telomerase activity.

The present study has also demonstrated that DSCs treated with NVP gradually lost their stemness characterizations. Most of the cell types, including MSCs generally express various CD markers which often act as receptors or ligands on their cell surface, and their level of cell expression is according to the cell type and their stage of differentiation, and over 300 CD markers have been constantly distinguished up to date (Lv et al. 2014). The several types of CD markers have thus used for identification and characterization of the MSCs. It has been showed that CD34 and CD45 surface markers are expressed at a very low level or not expressed, and CD90 and CD105 surface markers are highly expressed in the isolated DSCs, as other MSCs (Jeon et al. 2011b, 2015). Thus, the low expression of CD34 and CD45 cell and high expression of CD90 and CD105 surface markers for MSCs were considered as one of the main characterization criteria for defining DSCs (Dominici et al. 2006; Jeon et al. 2011b, 2015). However, the expression of CD34 and CD45 stem cell negative markers was gradually increased, and CD90 and CD105 stem cell positive surface markers were gradually decreased in the DSCs treated with NVP. Another important biologic characterization of MSCs is the differentiation capacity into cell types of mesodermal lineages, such as osteocytes, chondrocytes and adipocytes under lineage-specific induction conditions *in vitro*. Many previous studies have widely demonstrated that MSCs, including DSCs, can be easily differentiated into neuronal cells of ectodermal lineage as well as mesodermal lineages by exhibiting lineage-specific staining and positive expression for genes associated with relevant differentiated cell types (Jeon et al. 2011b, 2015). However, the present results have also shown that the differentiation capacity is gradually decreased in the DSCs after the NVP treatment, depending on the intensity of special staining for confirmation of differentiation. But, the previous study has demonstrated that cellular differentiation into myelomonocytic-like cell type is induced after the NVP treatment in cancer cells via morphological staining (Mangiacasale et al. 2003). Likewise, one study has also shown that the NVP treatment results in senescent-like abnormal morphology, such as more flattened shape and elongated dendritic-like extensions with reduced cluster formation in the human renal carcinoma (Landriscina et al. 2008). The alteration of flattened and elongated cell morphology was frequently observed in the cancer cells reached at senescent status by treatment of anti-tumor drug (Kim et al. 2017). It has been widely known that fully differentiated cancer cells are tended to be increased to cells with normal morphology and spreading growth, such as fibroblasts, compared to undifferentiated cancer cells (Busch et al. 2017). The present study has shown that the senescent-like flattened and elongated cell shape are usually observed in the cells treated with 500 μM NVP, in particular, as shown in Figure 1A. Thus, we assume that the alteration of cell morphology is thought to be a consequence of cellular damage and senescence by NVP treatment rather than cellular differentiation, and further the decreased differentiation capacity in the DSCs may be also due to the damaged gene expression by the NVP treatment. Any intrinsic gene expression related to cellular differentiation was not carried out and further studies will be needed for the investigation of differentiation capacity in the NVP-treated DSCs.

In the present study, the stemness characterizations related with pluripotency and self-renewability capacity of the cells was investigated in the DSCs treated with NVP. It has been known that expressions of NANOG, OCT-4 and SOX-2 transcripts are basic transcription factors for pluripotency in MSCs which are associated with their self-renewability and stemness (Rodgerson and Harris 2011). In our previous study, we have also demonstrated that these transcription factors were steadily expressed in the MSCs derived from dental tissues (Jeon et al. 2015). However, the expression level of NANOG, OCT-4 and SOX-2 transcription factors was gradually decreased when DSCs were treated with NVP. But, comparatively more pluripotency marker expression was found in NVP-untreated control DSCs. The function of RTase on the stemness and cellular differentiation in the MSCs is still unclear. Similar to our results, previous reports have also shown that the inhibition of RTase through chemicals, such as NVP or RNA interference, is induced to cellular differentiation with reduced cell growth in cancer cells showing the high activity of RTase (Mangiacasale et al. 2003; Sciamanna et al. 2005). Furthermore, totipotency in both mouse embryonic stem cells and the naive-like status in human embryonic stem cells was tightly related with the high expression of retrotransposon by high RTase activity (Yin et al. 2018). As mentioned above, inhibition of RTase activity can result in decreased gene expression by epigenetics modification, and decrease in the expression of these transcription factors, as shown in the present study, should be related with the loss of stemness by inhibition of RTase in the DSCs treated with NVP. Moreover, we also assume that the RTase in the stem cells might have an important role in cell differentiation as well as cell growth by altering gene expression.

In the present study, we demonstrated that RTase activity was related with basic cellular properties, such as cell growth and stemness characterizations in the MSCs derived from dental tissue. The exposure duration to NVP was 2 weeks in the present study; however, further studies are needed to investigate the effects on the stemness characterizations after more prolonged NVP exposure. And, the intrinsic mechanisms induced by RTase activity were not fully demonstrated in the present study and further in-depth studies will be needed to determine the fundamental biological functions and roles of RTase activity, possibly contributing to the regenerative medicine and stem cell research using MSCs.
